# Loot

**Published:** 1870-03

**Authors:** 


### Loot.

*On Earth-Closets.*

THE disinfecting property of dry earth, humus, clay, charcoal, peat, bone black, ashes, and other solid substances, has long been known, and all of these bodies have been applied in one way and another for the prevention of bad odors, for the filtration of water and absorption of gases. The bodies of the dead have, with most nations, been buried beneath the soil, and thus all danger likely to arise during their decay has been removed.

In the thickly populated regions of the East, necessity has impelled the inhabitants to have recourse to the same principle in the disinfecting of all fæcal matter, but in a sparsely settled country great carelessness is apt to creep in and to maintain its supremacy, long after a large increase of population imperatively demands as much care in the removal of all excremental matter as it does in the proper disposition of dead bodies.

The action of dry earth is not only chemical but physical, — and consists mainly in the absorption and removal of the water necessary to the decay of organic substances; the formation of dangerous gases is thus prevented and the animal matter is left to a slow decay (combustion) and no odors can arise.

A small amount of earth is sufficient to disinfect a considerable quantity of putrid or offensive matter, and for this reason its use has been strongly recommended in hospitals in cases of bad sores and wounds.

The recent learned researches of Pettenkofer on the causes of typhus and cholera have led to the conclusion that these dangerous diseases prevail in regions where the water from sewers, out-houses, sinks, etc., approaches near the surface. In fact the cholera was traced along the water courses, and the upper part of the same valley would be left untouched, while the lower portions were subjected to the ravages of the disease. During a dry season the disease was less likely to appear than during a wet. The incontrovertible conclusion forced itself upon the mind of Pettenkofer that there was nothing more dangerous than to have the water near a dwelling contaminated by the diseased fæcal excreta of the population, and he sought for some remedy for this startling evil. If he had heard of the epidemic in the National Hotel at Washington, and of the fever that proved so fatal at the Pittsfield Seminary, he would have had some further confirmation of the accuracy of this theory.

The labors of Pettenkofer in Germany have been ably seconded by those of the Rev. Henry Moule, of Fordington Vicarage, Dorsetshire, England. He experimented with a large number of substances, and finally returned to mother earth, where he found, in the cheapest of all material, the very best agent for the removal of danger.

He proposed to use dry earth as a disinfectant, and invented a simple contrivance for the application of this principle to commodes, thus doing away with all out-houses and other sources of evil about the homestead.

If his suggestions could be universally applied, there is no doubt that the death rate from typhus, intermittent fever and cholera, would be greatly reduced, and the general health of every family largely promoted. We propose to give a description of the apparatus, by the use of which the disinfecting property of dry earth can be practically applied in every household, and for this purpose shall make copious extracts from a pamphlet on the subject of earth-closets, by Mr. George E. Waring, late engineer of the Central Park, and published by the Tribune Association; a pamphlet, by the way, that ought to be circulated as a tract in every portion of the country.

"The principle of the earth-closet is based on the power of clay and the decomposed organic matter found in the soil to absorb and retain all offensive odors and all fertilizing matters; and it consists, especially, of a mechanical contrivance, (attached to the ordinary seat), for measuring out and discharging into the vault or pan below a sufficient quantity of sifted dry earth to entirely cover the solid ordure and to absorb the urine.

"The discharge of earth is effected by an ordinary pull-up similar to that used in the water-closet, or, (in the self-acting apparatus), by the rising of the seat when the weight of the person is removed. The vault or pan, under the seat, is so arranged that the accumulation may be removed at pleasure. From the moment when the earth is discharged, and the evacuation is covered, all offensive exhalation entirely ceases. Under certain circumstances, there may be. at times, a slight odor as of guano mixed with earth, but this is so trifling, and so local, that a commode arranged on this plan may, without the least annoyance, be kept in use in a room.

"Mr. Moule's patented invention consists of a tight receptacle under the seat, a reservoir for storing dry earth, and an apparatus to measure out the requisite quantity and throw it upon the deposit. A hopper-shaped reservoir, made of galvanized iron, is supported by a frame-work at the back of the seat. Connected with the handle, at the right hand side, there is an iron lever, which operates a movable box at the bottom of the reservoir and causes it to discharge its contents directly under the seat. When the handle is dropped, the box returns to its position, and is immediately filled preparatory to another use."

It is difficult, without the aid of diagrams, to convey an accurate idea of the apparatus employed, but any one sufficiently interested in the subject can easily obtain drawings or descriptions from the Earth-Closet Company of Hartford, Connecticut. After the galvanized iron scuttle is full it can be emptied in a protected place and the contents left to air dry, when it can be again used and again dried, as many as eight or ten times. It will be seen from this that a very small amount of earth is sufficient to serve the wants of a large family. As soon as the pores of the earth have become full it can be employed as s most valuable fertilizer. This brings us to speak of the important saving effected by such an application of the fæcal deposits. The value of composts annually wasted has been put at very high rates, and this view alone would warrant the adoption of the new system, even if the far more important sanitary reason were not enough to convince the most skeptical of its value.

Mr. Waring estimates the direct annual loss to the country by the waste of night soil at $50,000,000. The indirect loss in the falling off of the producing power of the land is left out of view, and the other far more serious loss of life occasioned by diseases brought on by open privy cannot be computed in money, but must especially attract the attention of every student of social science in the country.

There are not wanting statistics of experiments tried in England of employing the dried earth as a fertilizer, after it had been used in the closets, and the results were such that many farmers expressed their willingness to pay as much for the earth as they would for guano. It is probable that in course of time dealers in guano will be prepared to purchase the article at a fair price, and thus those families who have no garden will be able to sell night-soil enough to pay for the vegetables consumed at their table.

"The supply of earth to the house, is as easy as that of coals. To the closet it may be introduced more easily than water is supplied by a forcing pump, and to the commode it can be conveyed just as coal is carried to the chamber. After use, it can be removed, in either case, by the bucket or box placed under the seat, or from the fixed reservoir, with less offence than that of the ordinary slop bucket, indeed, (I speak after four years experience), with as little offence as is found in the removal of coal ashes, so that, while servants and others will shrink from the novelty, and at first imagine difficulties, yet many, to my knowledge, would now vastly prefer the daily removal of the bucket or the soil to either the daily working of a forcing pump, or to being called upon once a year, or once in three years, to assist in emptying a vault or cesspool.

"The earth after it has absorbed the excretions, is so perfectly inoffensive, that I have known some that had been mechanically mixed, taken the same day to London in a box in his carpet bag, by a chemist; and by two engineers' clerks, it was taken wrapped in brown paper in the side pocket." Mr. Moule).

Mr. Waring speaks of the difficulty of properly disposing of kitchen and other house slops and makes the following admirable suggestions:

"Any means by which they may be filtered through or covered with earth will solve the problem. The English plan is to run them through open-jointed draining tiles laid about a foot below the surface of the ground. The lines of tiles being within five or six feet of each other, great fertility may thus be given to the ground of a garden. In our colder states this plan would not be effective in winter. A large hogshead standing on the ground, the bottom being removed, will be serviceable at all seasons of the year, and will produce an immense amount of valuable manure.

"A wooden hopper provided with a lid, and extending to the middle of the hogshead, serves to catch all grease and garbage, and to conduct the liquid to a point where it has earth above and below it. In this way the kitchen slops of a family of five persons for four months were entirely disinfected without changing the earth, and nothing but pure water escaped at the bottom of the hogshead."

Professor Samuel W. Johnson, of Yale College, than whom we have no higher authority in all agricultural matters, writes to the New Haven *Palladium* in the following forcible language, in reference to the Earth-Closets:

"There are two grave questions which enforce attention from every dweller in the city, and should not be neglected by those who have the country for their home. These questions relate to the disposition of the liquid and solid waste of the human body. One of them is, how shall the waste be effectually prevented from being an annoyance and source of disease? and the other, how shall it be made a means of fertility to the soil, and thus an item of national wealth?

"Nothing is better established than the connection between human excrement and certain fearful epidemics.

"It is on all hands admitted that the cholera is most frequently and certainly transmitted to healthy persons, by the intestinal evacuations of those who have been sick with this disease.

"Typhoid fever, a form of disease very prevalent among us, is often traceable, with scarcely less certainty, to privy vaults, cesspools and sewers. It is stated that Prince Albert, of England, probably contracted the disease that was fatal to him from the foul air that found its way into his study out of a forgotten sewer, through a crack in the wall."

"Most often it is our drinking water that brings into us the contamination. In multitudes of cases the well is but a few yards or feet from a cesspool that receives the kitchen slops on one hand and a privy vault on the other. The writer knows a well which furnished good water for about five years after it was excavated, in what was until than a vacant lot, but after this interval became unpleasant in taste, its flavor plainly suggesting the nature of its impurities.

"In his researches of the cholera, in Bavaria, in 1854, Pettenkofer traced its spread in several cases, in the most indubitable manner, to the use of water which had been in contact with the fæces of cholera patients.

"The safest mode of escaping the evils in question, hitherto adopted in closely built towns, consists in removing all human excreta to a distance, by subterranean sewerage.

"The waste involved in the 'civilized' way of treating the materials under notice is immense. Every harvest brings from the country to the city, from the West to the East, a vast bulk of beef, corn and hay, whose use to the city-people does not, for the most part, consist in any permanent giving of its elements, but which, after having weighed the wheel of life through half a ton, and dropped off as waste, admits of conversion into food again, if but carried back to the fields. The gardeners and farmers in our immediate vicinity are obliged to disburse heavy sums each year for the phosphates and nitrogen which their crops demand, and which their land cannot adequately supply. The guanos and fish manures, which are brought from a distance or manufactured at heavy cost for their use, are in reality paid for, not by them, but by those who purchase their produce in the city markets. The animal who stands at the head of creation requires the richest food, and yields to the food producer the richest return It requires but little art to convert his excrement into manure, and the conversion may be made extremely profitable.

"The means of satisfying, at once, all demands of sanitary science and of agriculture is, however, fortunately everywhere at hand, and of extreme simplicity and cheapness in its application. *Dry and fine earth* is the material.

"This property of earth is no new discovery. Its use was prescribed to the Israelites, (Deuteronomy, xxiii, 12 and 13,) and is turned to good account by the instincts of our domestic carnivora. The Rev. Henry Moule, an English clergyman, was the first to elaborate, by a careful study of the subject, a plan for the systematic employment of earth for this purpose.

"The arrangements required to constitute an earth-closet are not necessarily complex or expensive. It is only needful that a space be had below the privy seat, the bottom of which should be of flagging or cement, and a little above the ground level, or at least protected from the wet of rain and of the ground. The space should communicate with a shed at the rear of the privy, to hold on one side a load or two of dry fine earth (not sand) or sifted coal ashes, and leave an equal room unoccupied on the other, For hospital or sick-room use, either a simple commode or pail, with a hod of earth to apply, or the self-acting commodes of Mr. Moule, may be used.

"Very important it is that hotels, schools, and, we may add, colleges, should be provided with this labor and health-saving arrangement. In large schools it is sufficient to put the application of earth in charge of an attendant. In hotels, the self-acting apparatus is better.

"The fertilizing value of the properly managed compost should be abundant renumeration to parties supplying earth, especially as its carriage is not attended with the slightest odor, and requires not the cover of darkness to mitigate its terrors, while its use is less disagreeable than that of Peruvian or fish guano, and not worse than the employment of any old compost.

"Reader, lose no time in providing yourself, and inciting your neighbor to provide, some form of earth-closet, in lieu of the vault which has hitherto sufficed. Health and economy both demand it! City authorities would do well to enact that all privies, within a hundred feet of dwellings, or of wells in use, should be converted into earth-closets, and to provide for their systematic and thorough inspection."

The advantages and importance of earth-closets must be apparent to every one who has followed us thus far in our article. They require very little architectural change, and can be put anywhere when a vault is not already dug; they are entirely without odor, and preserve in the best way the full agricultural value of the manure.

If there is a suitable room about the premises for the construction of the reservoir for the earth, it can be made sufficiently large to require to be rarely filled. Where there is a scarcity of earth it is better to dry and use it several times. The contents of the hod must, in this event, be carried daily to a particular spot, and there dried and sifted. In warm climates this can easily be done, but in wet seasons some artificial method for drying must be employed. Twenty-four cubic inches of earth is probably enough for each drop, or a cubic foot for seventy-two times; at the rate of 400 visits per annum for each person, the yearly consumption would be five and a half cubic feet of earth, and, where the earth is used over 8 times, the requirements would be about one cubic foot per annum for each person. This estimate shows that the labor of providing all of the necessary earth must be very small in the country, and it is probable that, after a few years, the rich compost will become a source of profit, and the earth will cost nothing.

Surely a system that will save many lives, keep up the good health of the family, avoid waste of valuable manure, and is cheap and easy of application, ought to be universally substituted for the wasteful, dangerous, unhealthy custom handed down to us by our forefathers. — *Journal of Applied Chemistry.*

### *Hydrate of Chloral.*

FROM the *Journal of Applied Chemistry* we extract:

"Doctor Bouchut confirms the observations made by Liebreich. The contradictory opinions expressed in reference to the action of chloral are due to the impurity of the material employed. With the hydrate of chloral pure, the results are rapid, evident, energetic. They are hypnotism (sleep) the most tranquil, and sometimes an absolute insensibility. The liquid chloral ought never to be employed. The solid hydrate ought always to be taken for solution in any convenient agent. The acicular crystals, or the white snowy mass, can generally be assumed to be pure; but, to make sure of the point, it is well to test it with a concentrated solution of potash. If the hydrate of chloral is pure, it will color the potash to a scarcely appreciable faint yellow tinge, and disengage a distinct odor of chloroform, and sometimes the liquid will remain perfectly colorless. If the liquid becomes brown, and disengages the vapors of chloroform, mingled with chloracetic acid, it is a proof of impurity. The liberation of acrid, disagreeable gases, is another proof of impurity. * * * *

"At the meeting of the Chemical Society of Berlin, October 25, 1869, Messrs. Mueller and Paul presented a paper on the preparation of hydrate of chloral. They pass chlorine gas through absolute alcohol until the entire contents of the flask is converted into a solid, white, crystalline mass. For this purpose a strong current of dry chlorine gas must be passed through the alcohol for 60 to 70 hours. If the operation be conducted with care, a uniform product of nearly pure hydrate of chloral will be obtained. The hydrate can be purified by sublimation; for this purpose two funnels are employed, the lower one passes into the mouth of the flask, and the upper one is inverted upon it; the sublimed crystals collect on the walls of the two funnels, and can be easily scraped off, and yields a snow-white, dry, neutral crystalline powder. The novelty of the process consists in converting the whole of the alcohol into the hydrate of chloral, and the subsequent purification by sublimation."

The *Lancet and Observer* gives a translation from the *Bulletin de Therapeutique*, Nov. 26, 1869, of which the following are the conclusions:

"1. The hydrate of chloral is a powerful sedative of the motor and sensitive nervous system.

"2. If the hydrate of chloral is not very pure, so that by the addition of potassa to a solution, the vapors of chloroform are liberated without discoloration of the liquid, it is without action, and may be very dangerous.

"3. Hydrate of chloral should not be administered to the adult in doses exceeding seventy-five to ninety grains, and in children it is necessary to commence with fifteen to thirty grains.

"4. Preparations of hydrate of chloral should not be prepared too long previous to administration, for they may change and lose their efficacy.

"5. Hydrate of chloral may be given by the mouth, or in enemas, which produce the same effect as when administered by the stomach; but the latter method is preferable.

"6. Hydrate of chloral should not be given to persons affected with organic disease of the heart or brain.

"7. It is by the production of chloroform in the blood, under the influence of its alkaline reaction, that ingested chloral causes sleep and anæsthesia.

"8. It is dangerous in man to administer hydrate of chloral by subcutaneous injections.

"9. Under the influence of hydrate of chloral the arterial tension is augmented, and the frequence of the pulse is somewhat increased; and this tension diminishes on awaking, as is shown by sphygmographic traces.

"10. The urine of chloral sleep is neutral, and boiled with Fehling's liquor does not at first reduce the salts of copper, but the next day, when we find chloral passed by the kidneys, the urine is more dense, and reduces the salts of copper, so that we may be led to think there is a glycosuria, which in fact does not exist.

"11. Hydrate of chloral seldom causes vomiting, and never purging.

"12. The temperature is slightly lowered by medicinal doses of hydrate of chloral, and it should, therefore, be classed as a cooling remedy.

"13. Under the use of hydrate of chloral, cutaneous perspiration is diminished, and the skin is somewhat dryer than is normal.

"14. Hydrate of chloral has this advantage, that we can regulate the dose necessary to produce anæsthesia, while in administering chloroform we cannot control precisely the quantity of vapor; when we employ chloroform we are not exactly sure of what we are doing, and it is this that renders it dangerous.

"15. The action of hydrate of chloral is exactly that of chloroform, but it is more slowly produced, and lasts much longer.

"16. With some persons under the influence of chloral there is a muscular and moral agitation resembling alcoholic intoxication, but this intoxication presents nothing disagreeable or disgusting.

"17. In nearly every case the sleep is characterized by well pronounced anæsthesia, and seldom accompanied by hyperesthesia.

"18. The degree of anæsthesia is proportionate to the dose employed. In the dose of thirty to seventy-five grains, according to the age, it is complete, and permits the application of Vienna paste cauteries without pain, or even the extraction of teeth.

"19. Compared with opium, which causes vomiting, which destroys the appetite, which heats and stimulates, which constipates, which excites perspiration, which produces sleep slowly and heavily, which leaves, on awaking, distress and prolonged somnolence, the hydrate of chloral neither vomits nor constipates, and improves the appetite; it lessens perspiration, and cools the surface; it causes quick and prolonged sleep, and on awaking leaves neither somnolence nor hebetude of mind, and can be taken several days in succession.

"20. In large doses, hydrate of chloral produces algidity, while opium, on the contrary, causes heat and diaphoresis.

"21. We may repeat the dose of forty-five to seventy-five grains two or three times during the day, without inconvenience, and with the result, each time, of several hours' sleep, separated by a short period of wakefulness.

"22. Therapeutically considered, the hydrate of chloral subdues the violent pains of gout, and the intense sufferings of renal colic, or large burns and toothache. It is, in a word, the first of anæsthetics administerable by the stomach.

"23. In cases of accouchement in which we desire to resort to chloroform, hydrate of chloral may be substituted, to appease normal pains, to facilitate obstetrical operations, and to combat eclampsia.

"24. It is the most prompt and efficacious remedy, in intense chorea, when we wish quickly to suspend an agitation which menaces the days of the patient."

### *Partial Paraplegia.*

In a case, now under our care, of partial paraplegia, probably resulting from injury of the spine by a fall a year or two since, but wherein also there had been some fears of a *specific* cause. The patient has previously consulted Ricord and Brown-Sequard in Paris, and Pavy in London. Ricord diagnosed the case as non-specific. Brown-Sequard prescribed as follows:

| | | | |
|---|---|---|---|
| (1.) | ℞ | Potassii iodid., - - - - - | ʒ ij; |
| | | Ammon. iodid., - - - - - - | ʒ iij; |
| | | Ammon. carb., - - - - - | ℈ ij; |
| | | Infus. columbæ, - - - - - - | ℥ v; |

M. S.—Two tablespoonfuls, before meals, with a little water.

| | | | |
|---|---|---|---|
| (2.) | ℞ | Extr. belladonnæ, - - - - - | gr. iv; |
| | | Secalis cornuti pulv. recentis, - - | ʒ j; |

M. ft. pil. xxx.
S.—One pill after each meal.

| | | | |
|---|---|---|---|
| (3.) | ℞ | Ammon. bromid., - - - - - | ʒ ij; |
| | | Aq. destillatæ fl., - - - - - | ℥ v; |

M. S.—A teaspoonful and a half twice a day, before meals. Indorsed: (This can be mixed with the first.)

On the second (2) formula is indorsed:

(After two weeks begin with this in any case, and use for two weeks. In case it does not produce a good effect, try the third formula a month at least.)

Dr. Pavy prescribed:

| | | |
|---|---|---|
| ℞ | Pot. bromid., - - - - - - | ʒ ij; |
| | Pot. iodid., - - - - - - - | ʒ j; |
| | Sp. ammon. ar., - - - - - - | ℥ j; |
| | Aq. camph. ad., - - - - - - | ℥ xij; |

Misce fiat mist; cap. cochl ij magna ter quotidie.

℞ Extr. belladonnæ, - - - - - - gr. ¼;
Extr. gentianæ, - - - - - - gr. iij;
Misce ft. pil. et sep. ad xij, cap. j ter quotidie.

Two weeks later he prescribed:

℞ Pot. iodidi, - - - - - - ℈j:

Tinc. nucis vomicæ, - - - - - - ʒ iss;

Ext. taraxaci, - - - - - - ʒ iij;

Aq. destiil. ad., - - - - - - ℥ vj;

Misce fiat mist. cap. cochl. ij magna ter quotidie.

The general coincidence of the independent prescriptions of these two eminent gentlemen, is a proud vindication of trans-atlantic skill in diagnosis, and "the tendency of great minds to run in the same channel." It is also a tribute to the general power of the medicines employed, in Europe as well as America. We are personally gratified to find that they are not yet entirely superseded by iodoform, carbolic acid, or the hydrate of chloral.

### *Paste.*

THE following is a recipe for making common paste which will keep a long time without fermentation:

Dissolve an ounce of alum in a quart of water warmed; when cold add as much flour as will give it the consistence of cream; then strew into it as much powdered rosin as will lie on a shilling, and two or three cloves, ground. It will keep for a year, and when dry may be softened with water.

### *Morphine vs. Atropine.*

DR. MINER (*Buffalo Medical Journal*) reports a case of morphine poisoning apparently relieved by atropine.

### *Kerosene.*

SEVEN out of sixty-four fires in New York during the month of August, was caused by kerosene. Out of one hundred samples of oil tested by the Board of Health, only *one* came up to the legal standard. Manufacturers and dealers are reckless in their violation of the law, and will continue to be so until public sentiment demands its rigid enforcement. The crime ought to be punished as severely as arson.—*Boston Journal of Chemistry.*

The daily accidents, often of a most fearful character, which occur from the use of this substance, aside from the mere risk of property, would seem to demand the most stringent legislation upon this subject.

### *Alcohol on Sponge.*

J. B. HOUGH, M.D., of Ridgeville, Ohio, says: Knowing the fact that absolute or *strong* alcohol will quickly *set* the fibres of common sponge, after having been moulded or compressed into any given size or shape, I was led to the following quick and easy method of preparing sponge tents, tampons, etc.:

The sponge is first thoroughly moistened with water and pressed as dry as the strength of the hand will permit; then having formed it into the desired shape and size by the hand, or by pressing into a quill or any other tube or mould, it is immersed into the alcohol. If the spirit is sufficiently strong (90 to 100 per cent.), the sponge is *immediately* set into the given shape, which it retains perfectly after the pressure or mould is removed. It is then hard, firm and inflexible, and may be trimmed to a sharp point or any other desired shape.

To restore it to its former size and shape, it is only necessary to moisten it with a few drops of water. The alcohol sets the sponge perfectly, whether the amount of compression be much or little, so that the degree of dilatation attainable by the use of tents thus prepared, will of course depend upon the size after moulding, and the degree of pressure used. As this process of preparation works perfectly and *without delay*, its advantages are obvious.—*Boston Medical and Surgical Journal; Cincinnati Lancet and Observer.*

### *The Perils of Fashion.*

We are indebted to a lady correspondent for the correction of a mistake, or rather an omission, in our recent article on Tight-lacing. In ascribing the ungainly, feeble, and tottering walk of our modern fine ladies and their middle-class imitators to the decrepitude induced by tight-lacing, we omitted to mention another fashionable folly which assists in the production of this evil, and has also other sins of his own to answer for. The custom of wearing high boot-heels, and those too so much smaller than the actual heel of the wearer as to afford no solid support, but only a balancing-point, is a source of much mischief. In the first place, it throws the centre of gravity of the body so far forward that a free and gracefully erect carriage is impossible. Secondly, there being no firm support to the heel, ladies are very apt to twist the ankle suddenly by overbalancing themselves; and this is not only bad in itself, but the fear of its occurrence makes them assume a timid, mincing gait. And, thirdly, the effect of driving the foot constantly forward into the toe of the boot, is to produce a very ugly and painful distortion of the great toe joint.

There is little need for wonder at the almost fierce contempt with which young men whose characters are at all above the lowest grades of conventional inanity regard the average "girl of the period." It cannot be denied that there is a significant correspondence between the æsthetic hideousness and the degrading effects on physical health which are produced by tight stays and crippling boots, and a certain mental and moral tone in female society of the present day, which is no less surprising than it is repulsive. The whole dress and carriage of our fashionable women, for several years past, has been modelling itself, with less and less concealment, upon the ideal furnished by Parisian *lorettes* of the consumptive Traviata type. It is not our business to set up as moral censors. But we may be excused if, for once in a way, we find it impossible to ignore the logical though repulsive consistency of the *grandes dames* and the citizenesses who are willing to spoil their lungs and their digestions, and endanger their chances of happy maternity, for the sake of a wasp waist; to talk slang closely verging on indecency, for the sake of the tenth part of a chance of catching a husband; and to simper and leer at the indecencies of a *Grande Duchesse de Gerolstein*, in order to escape the dreaded imputation of a deficiency in *chic.*—*Lancet*, Oct. 2, 1869.

***Dissection Wounds.***

THE name of Professor Boehm of Berlin, must be added to the long list of victims who have perished in the accomplishment of the perilous duties of the profession. While dissecting a body in the presence of the students, he scratched his hand with the scalpel, but so slightly that he neglected to cauterize the wound or to take any other precaution. Two days afterwards, the hand had swollen enormously, and since then all the means which had been tried to arrest the progress of the disease have been to no purpose.—*Medical News.*

***Remarkable Operation — Extirpation of a Kidney.***

AT a meeting of the New York Medical Journal Association, Dr. H. Knapp made a communication concerning a new triumph of surgery, namely, the *successful extirpation of a kidney*, by Prof. G. Simon, Heidelberg. The patient on whom the operation had been performed, early in August last, was a woman both of whose ovaries and part of the uterus had been excised previously by another surgeon, for ovarian dropsy. The wound had healed, but a *urinary fistula* had established in the cicatrix of the abdominal wall. By ingenious experiments, Prof. Simon found out that it was not a piece of the bladder, but one of the ureters, and which of the two, that had been implicated in the cicatrix. The extirpation of the corresponding kidney was not easy, but effected without a serious accident. The patient experienced some fever during the first two weeks after the operation, but recovered entirely within six weeks. Dr. Knapp said that he felt happy in being authorized to give the Society this preliminary notice, according to a letter he had just received from

Prof. Simon himself. He thought this operation would interest the Society, not only as the newest, but also as one of the most remarkable surgical achievements.—*Phil. Medical and Surgical Reporter.*

### *Water in Alcohol.*

A SIMPLE test of the percentage of water in alcoholic solution is furnished by the affinity of alcohol for chloroform. In the experiment a little chloroform is poured into a graduated glass tube, and a given volume of the spirits to be tested is added, and both well shaken up together. On settling, all the water will be found to have separated from the alcohol (which has combined with the chloroform), and its amount can be read off on the graduated tube.—*American Artisan.*

### *Bromide of Potassium for the Troubles of Teething.*

THE bromide of potassium is highly spoken of by Dr. Hoehling (*St. Louis Med. Jour.*), and by Dr. Caro (*Medical Record*), as a remedy for faulty nutrition, irritability of the stomach, vomiting, restlessness, diarrhœa, etc., resulting from teething. From half a grain to a grain, given every hour or two, will, in a short time, often procure relief and sleep when other means have failed. The following is selected from Dr. Caro's list of twenty cases: S. Fay, nine months old, has six teeth, nursed by a healthy mother. After fifteen days of diarrhœa, with from fifteen to twenty passages every twenty-four hours, was cured by a mixture of one drachm of bromide of potassium in an ounce of mucilage, twenty drops every hour. Dr. Caro adds: "I have had fifteen similar cases in every form, having been sick from fifteen to twenty days before coming to me, all treated and cured by the bromide of potassium."

